# Smart Pothole Detection Using Deep Learning Based on Dilated Convolution

**DOI:** 10.3390/s21248406

**Published:** 2021-12-16

**Authors:** Khaled R. Ahmed

**Affiliations:** School of Computing, Southern Illinois University, Carbondale, IL 62901, USA; Khaled.ahmed@siu.edu; Tel.: +1-618-453-6057

**Keywords:** deep learning, smart potholes detection, YOLOv5, faster R-CNN

## Abstract

Roads make a huge contribution to the economy and act as a platform for transportation. Potholes in roads are one of the major concerns in transportation infrastructure. A lot of research has proposed using computer vision techniques to automate pothole detection that include a wide range of image processing and object detection algorithms. There is a need to automate the pothole detection process with adequate accuracy and speed and implement the process easily and with low setup cost. In this paper, we have developed efficient deep learning convolution neural networks (CNNs) to detect potholes in real-time with adequate accuracy. To reduce the computational cost and improve the training results, this paper proposes a modified VGG16 (MVGG16) network by removing some convolution layers and using different dilation rates. Moreover, this paper uses the MVGG16 as a backbone network for the Faster R-CNN. In addition, this work compares the performance of YOLOv5 (Large (Y_l_), Medium (Y_m_), and Small (Y_s_)) models with ResNet101 backbone and Faster R-CNN with ResNet50(FPN), VGG16, MobileNetV2, InceptionV3, and MVGG16 backbones. The experimental results show that the Y_s_ model is more applicable for real-time pothole detection because of its speed. In addition, using the MVGG16 network as the backbone of the Faster R-CNN provides better mean precision and shorter inference time than using VGG16, InceptionV3, or MobilNetV2 backbones. The proposed MVGG16 succeeds in balancing the pothole detection accuracy and speed.

## 1. Introduction

**Figure 1:**
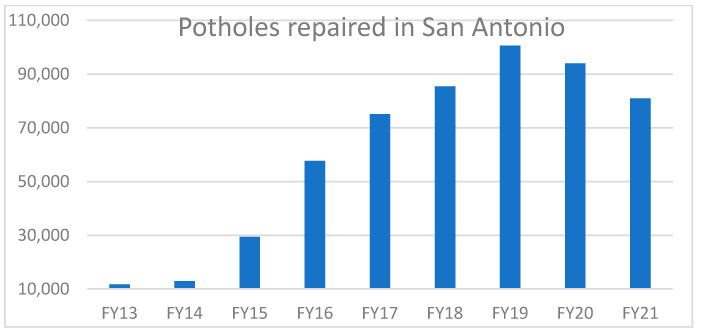
Annual number of potholes repaired From FY13 to FY21 in San Antonio, TX, USA drawn from data in [4,5].

Roads make a huge contribution to the overall of growth of an economy. Roads paved with asphalt, concrete, or both are widely used throughout the world as a platform for transportation. Road conditions include various types of defects such as potholes, unevenness of manholes, crack skid resistance, etc. Potholes can form because of low-quality materials, bad design that allows surface water accumulation, formation of ice in the cracks, etc. [1]. Every year potholes cause a lot of damage to life and property. Two-thirds of Americans are directly impacted and frustrated by potholes [2]. Since 2011, for five continuous years, motorists spent over $3 billion on vehicles to repair damage due to potholes. This cost approximately $300 on average for each driver. A report of India Economic Times in 2018 states that 3597 deaths due to potholes in roads were reported by the Supreme Court [3]. This is a huge toll, and the report claims that there are many cases that are unreported. Figure 1 shows the annual number of potholes repaired in city of San Antonio, Texas from fiscal year 2013 to 2021. It shows that the San Antonio pothole patrol crews repaired approximately 100,520 potholes in 2019 and 80,937 potholes in 2021 due to COVID-19 [4,5]. In Chicago there were over 156,000 potholes filled just in 2021 [6]. These numbers are relatively large amounts for cities. The road network of the United States is very large: a study from the Bureau of Transportation Statistics showed that there are approximately 746,100 miles of road in United States [7]. Keeping track of this length of road is a tedious task and almost impossible with the use of only human manpower. Integrating an automated pothole detection technique in vehicles would help to locate potholes and, accordingly, it will warn motorists and plan repair tasks.

There are various research efforts to automate the pothole detection process in roads using different approaches: sensor-based techniques [8,9,10,11,12], 3D reconstruction techniques (laser-based [13,14,15] and stereo vision-based [16,17,18,19,20]), image processing techniques [21,22,23,24,25,26,27,28], and model-based (machine-learning techniques and deep learning techniques) [29,30,31,32,33,34,35,36,37]. Senor-based techniques use vibration sensors to detect potholes. The accuracy of detecting potholes may be affected by false positive and false negative readings due to the vibration sensor detecting joints in roads as potholes or not detecting potholes in the center of a lane, respectively. The 3D reconstruction techniques collect 3D road data for pothole detection. They require costly configuration and computational efforts to reconstruct pavement surface and might suffer from cameras misalignment that could impact detection accuracy. Although traditional image processing techniques for pothole detection provide significant accuracy, they also need to perform challenging tasks such as extracting features manually and adjusting the image processing parameters and steps for different road conditions. The development of advanced image processing techniques and the availability of low-cost camera devices have motivated the development of model-based pothole detection techniques. Traditional machine learning (ML) techniques were applied to generate a trained model to detect potholes in 2D digital images. They achieve significant accuracy while utilizing high computational power. In addition, to improve the accuracy performance of ML techniques to detect potholes, experts are needed to manually extract features. Deep learning (DL) techniques used deep convolutional neural network (CNN) operations that are able to simultaneously automate the processes of features extraction and classification. One-stage detectors and two-stages detectors are two types of DL object detectors [20]. Several research efforts have been published to detect potholes that are one-stage detectors [33,34,35,38,39] such as You Only Look Once (YOLO) [40] and Single Shot Multibox Detector (SSD) [41]. They achieve moderate accuracy and fast detecting speed. However, few research efforts have been published to build two-stage detectors [42] to detect potholes such as Faster R-CNN [43]. They achieve high accuracy with slow detecting speed. Therefore, the main aim of this paper is to fill this gap in the literature by addressing the trade-off between accuracy and real-time performance. The main contributions of this paper are summarized as follows: (1) we proposed a dilated deep CNN as backbone for Faster R-CNN that increases the receptive field (R_F_) and reduces the number of calculations; (2) we developed a DL algorithm that generates a trade-off model that involves and balances the cost (inference time) against the benefits (accuracy) of potholes detection; (3) we developed and tested the YOLOv5 models for detecting potholes; and (4) we compared the performance of the proposed algorithm with the state-of-the-art methods.

The paper is organized as follows: a literature review is briefly discussed in Section 2. Section 3 comprises the theoretical background of deep learning algorithms used and the proposed modified VGG16. The experimental setup, dataset, and results are presented in Section 4. We conclude the paper with possible further enhancements in Section 5.

## 2. Related Work

Vision technologies provide efficient alternatives to automate tasks in various engineering fields such as transportation [44,45], agriculture [46,47], and industrial sectors [48,49,50]. This section illustrates some of the research efforts that have been developed to automate pothole detection in roads. The pothole detection techniques are classified into four approaches: sensor-based techniques, 3D reconstruction techniques (laser-based and stereo vision-based), image processing techniques, and model-based techniques (machine-learning and deep learning).

### 2.1. Sensor-Based Pothole Detection Approaches

There are multiple research efforts to detect potholes using various vibration sensors (such as ICP accelerometer or PC-oscilloscope) mounted to motorcycles, vehicles, and buses [8,9,10,11,12] to collect accelerated data to estimate pavement surface conditions. The vibration sensors could be built-in or [11] external to a PC. Eriksson [10] used GPS sensors and 3-axis accelerometers to collect data and used a machine-learning approach to identify severe road surface irregularities and potholes from accelerometer data (e.g., input *x* and *z* axis acceleration and vehicle speed). Five consecutive filters were studied: *z*-peak, *xz*-ratio, speed, high-pass, and speed vs. *z* ratio. These filters were used as well to exclude the generated data from events such as crossing railways and door slamming. To reduce the number of features, researchers used backward and forward selection, genetic algorithm, and support vector machine using principal component analysis [51]. Sensor-based pothole detection methods are not efficient techniques because: (1) they are not suitable to be implemented on devices with limited hardware [11], (2) they may suffer from false positives as the joints of road could be detected as potholes and false negatives as the potholes in the center of a lane cannot be detected because they are not hit by any of the vehicle’s wheels [10], (3) they cannot detect potholes until the vehicle pass over them, and (4) they lack information about the area and shape of potholes.

### 2.2. Three-Dimensional (3D) Reconstruction Pothole Detection Approaches

The 3D reconstruction approaches are categorized based on the technology used: laser-based or stereo-vision based techniques. The 3D laser scanner utilizes reflected laser pulses to create accurate digital models of objects [13,14,15]. These lasers could be used to detect potholes depth in real-time. Yu and Salari proposed a method [14] that involves the use of a light source to project a pattern of laser beams on the pavement, a camera to capture the pavement illuminated with the laser beams, and image processing on the captured images to identify potholes. Different approaches such as *Multi-window Median* filtering, *Tile Partitioning* with common thresholding [52], *Laser line* deformation, and *Template matching* were explored. The laser-based pothole detection techniques can detect potholes in real time. However, the cost of a 3D laser scanner is still expensive to mount on vehicles. Stereo vision techniques are used to extract 3D information from digital images. There are multiple research efforts using stereo vision methods to evaluate pavements and detect potholes [16,17,18,19]. Hou et al. [19] and Staniek [16] used two cameras to collect digital images. Zhang et al. [17] used a stereo camera to capture the left/right images of potholes. They calculated a disparity map using a computationally efficient algorithm. A surface fitting algorithm developed using low computational bi-square weighted robust least-squares method [53,54] were used to determine road surface and potholes. This pothole information was saved with geometric coordinates that can be used later to access the properties such as size and volume of potholes to prioritize the repairs accordingly. Like laser-based techniques, the stereo-vision techniques are also expensive in terms of configuration. Stereo-vision methods are not efficient because they (1) require a high computational effort to reconstruct pavement surface. (2) are vulnerable to vehicle vibration and camera misalignment that may affect the quality of the outcome.

### 2.3. Image Processing Pothole Detection Techniques

The image processing object detectors are dependent on hand-crafted representations to extract low-level features. There were several previous image-processing research efforts to detect potholes in a single image/frame [21,22,23,24], and other video-based methods were proposed to detect potholes and count their number over a series of frames [21,25,26,27,28]. The authors in [24] collected different frames and converted the frames into blurring grayscale images and then applied morphological and edge detection methods [55] to identify contours that are run through a Hough transform algorithm to extract features. Ouma et al. [56] applied fuzzy c-means clustering algorithm and morphological reconstruction techniques to 2D color images to detect potholes on asphalt pavement. In addition, Nienaber et al. used image processing to identify the potholes on roads and reject unwanted objects such as vehicle and plants from the image [22]. Frames are processed by simple image processing techniques such as Canny filters [57] and contour detection to locate potholes. The experiments resulted in precision of 81.8% with recall of 74.4%. Although the accuracy values are satisfactory in the test images, it is not guaranteed that using the same techniques in all type of roads will result in the same accuracy. The authors in [58] detect potholes in three stages: (1) pre-processing to extract the dark areas from a grayscale image, (2) candidate extraction to find the vanishing point to create virtual lanes, and (3) cascade detector to extract the pothole region using some threshold values. This technique achieved 88% accuracy with recall of 71%. Similarly, in [59], the authors detect potholes in three stages: (1) segmentation using histograms and morphology filters to extract dark regions, (2) candidate region extracted using various features, such as size and compactness, and (3) decision making as to whether candidate regions are potholes through comparing pothole and background features. The detection’s accuracy of the potholes using image processing approaches will be affected by the road conditions such as existence of dirt on the road and the variation in the pothole size. Thus, these approaches required adjusting the image processing parameters and steps for different road conditions, which are tedious tasks. In addition, these approaches are not suitable for real-time potholed detection because they require high computational power due to their computational complexities.

### 2.4. Model-Based Approaches for Potholes Detection Techniques

There is an increasing tendency of applying machine learning (ML) methods to generate trained models to detect potholes in 2D digital images. Support vector machine (SVM) was used as a ML algorithm for road information analysis and pothole detection [29]. Texture measure based on histograms was used as the feature of the image and non-linear SVM was used to detect whether the image includes potholes. The authors in [30] created a SVM trained by a set of scale-invariant feature transform (SIFT) features for recognizing potholes in labeled images. These methods achieved accuracy of 91.4% for detecting potholes. Hoang [31] used least squares SVM and neural network with steerable filter-based feature extraction and achieved a pothole detection accuracy rate of roughly 89%. Recently, Hoang et al. [32] integrated the SVM and the forensic-based investigation (FBI) metaheuristic to optimize the detection accuracy, and their experiments achieved an accuracy of 94.833% for detecting potholes. The stated machine learning approach achieved significant accuracy, although they encountered the following challenges: (1) manual feature extraction must be performed by experts to improve the accuracy performance during the pothole detection process, and (2) they required high computational power, which are not feasible to be used by drivers in their devices. Deep learning (DL) approaches provide an alternative solution that automatically processes features extraction and classification simultaneously through convolutional neural network (CNN) operations.

Recent studies used object detection DL to detect potholes accurately with significant speed. DL object detectors were classified into two categories: one-stage detectors and two-stages detectors [60]. The one-stage detector is a regression that implements a unified architecture to achieve results directly. The two-stage detector is based on selecting the region of interest and then detecting/classifying each region into various object classes. Several research efforts developed the one-stage detectors to detect potholes as follows. Maeda et al. [33] trained the model using the SSD-InceptionV2 and SSD-MobileNet frameworks. They installed the model on a smartphone, and their experiments showed recalls and precisions greater than 75% with an inference time of 1.5 s. Silvister et al. used SSD deep learning algorithms to detect potholes on a smartphone [38]. They validated the SSD detection against the detection done by sensor reading to reduce the false positives and have a backup mechanism if one of them fails. The authors claimed 96.7% detection accuracy. Similarly, the authors in [34] combined vision and vibration sensor-based methods for pothole detection. They used an accelerometer and the camera of a mobile phone for this task. Based on SSD with MobileNet, they were able to detect potholes with 55% accuracy for the sensor-based method, and 60% for the vision-based method. Song et al. [35] also used smartphones to gather movement information and the InceptionV3 [39] classifier to detect potholes. In addition, Redmon et al. developed YOLO, a one-stage object detector in 2016 [40]. YOLOv2, YOLOv3, and YOLOv3 Tiny have been applied to detect potholes [36]. The YOLOv3 Tiny and YOLOv4 achieved 76% and 85% high precision, respectively, and 49.71% and 85.39% mean average precision mAP@0.5, respectively [37]. The processing speed of both YOLOv3 and YOLOv4 is approximately 20 FPS (frames per second). A few research efforts developed two-stage detectors to detect potholes. The authors in [42] developed Faster R-CNN having 10 layers: 3 convolutional layers, 3 max-pooling layers, and 4 fully connected layers. They compared Faster R-CNN with YOLOv3 and SSD and concluded that the YOLOv3 model is faster than both SSD and Faster R-CNN model and YOLOv3 has the best accuracy of 82% [42]. Moreover, several research efforts [61,62,63] conclude that a two-stage detector such as Faster R-CNN always has a better precision rate with a lower speed compared to a one stage-detector such as YOLOv5. Balancing the potholes detection accuracy and processing (inference) time is needed. Thus, in our work we will fill this gap in the literature by addressing the trade-offs between accuracy and real-time performance by developing a novel DL algorithm that balances the pothole detection model’s accuracy and inference time. Moreover, the stated research works have shown acceptable levels of precision and inference time, but there is still room for improvement. Table 1 lists the limitations of the pothole detection approaches. Thus, this paper develops supervised DL algorithm to detect potholes in roads with significant accuracy while achieving real-time requirements.

## 3. Materials and Methods

There are two major categories of deep learning object detectors: two-stage detectors and one-stage detectors [60]. Two-stage detectors, in the first stage, generate region proposals from a region proposal network (RPN) that proposes bounding boxes that have the probabilities of having an object. The second stage contains an RoI pooling operation that extracts features from the bounding boxes generated by the RPN for classification and the bounding-box regression task. Faster R-CNN [43] is an example of a two-stage detector. One-stage detectors such as YOLO (You Only Look Once) [40] and SSD (Single Shot Multibox Detector) [41] are regression models that predict both bounding boxes and classification probabilities simultaneously without the region proposal step. Thus, two-stage detectors achieve high accuracy in terms of object localization and recognition whereas one-stage detectors are popular for their speed [64]. Backbone networks extract the features from input images and produce feature maps. Layers in the backbone network can be used for object detection as well as classification. The deeper the backbone, the more the features enhance the accuracy. Predefined backbones such as VGG16 [65], ResNet50 [17], and Darknet [66,67] are widely used in object detection algorithms as a means of feature extraction and classification. This paper proposed a modified version of VGG16 (called MVGG16) that generates high quality training results and reduces the required computation cost to detect potholes. The proposed MVGG16 is used as a backbone network to the two-stage detector (Faster R-CNN). The following sections briefly discuss the YOLOv5 [68] and Faster R-CNN [43] architectures and illustrate the architecture of the proposed MVGG16.

### 3.1. Faster R-CNN

Faster R-CNN [43] is one of the widely used two-stage detectors for object detection. Unlike YOLO, Faster R-CNN has two networks: a region proposal network (RPN) for generating region proposals and a classifier network for classifying the objects in the generated region proposals as shown in Figure 3. Anchors are the basic components of this architecture. Anchors are basically boxes and at each position in an image there are nine anchors by default. The default size for anchors is 128, 256, and 512, but this can be overridden. This paper used anchors with various sizes as shown in Table 2. The input images are passed through a CNN that generates a feature map. The next stage is the RPN, which finds a predefined number of regions from the feature map. With a list of possible objects and their locations represented by the bounding boxes, the Faster R-CNN uses a classifier to classify whether the bounding boxes contain desired classes of objects. The CNN used for feature extraction are also known as backbone networks. VGG16 [65], MobileNet [69], ResNet [17], etc., are few of the widely used backbone networks. In this paper, we proposed a modified VGG16 network as shown in Section 3.2 and Figure 3 by removing some convolution layers and using different dilation rates to reduce the computational cost and improve the training results. In addition, we have compared the performance of ResNet50 with feature pyramid network (FPN) [70], VGG16, MobileNetv2, Inception V3, and modified VGG16 to figure out which one is the best in terms of speed and accuracy. As shown in Figure 3, the input image is given to the backbone (e.g., VGG16) that processed it until the last convolution layer (except the last pooling layer). Each region of interest (RoI) pooling layer then produces a fixed-length (*H × W*) feature vector from the feature map (e.g., VGG16, *H = W* = 7). The generated feature vector is given to fully connected layers (Fc6 and Fc7). They then branched to two sibling output layers. The first sibling layer generates softmax probability of K object classes plus a “background” class. The second sibling layer produces four real-valued numbers (bounding box positions) for each of the K object classes. The Faster R-CNN multitask loss function is defined as follows [43].

(1) $$L\left(\left\{p_{i}\right\},\left\{t_{i}\right\}\right)=\frac{1}{N_{cls}}\underset{i}{\sum }L_{cls}\left(p_{i},{\bar{p}}_{i}\right)+\lambda \frac{1}{N_{reg}}\underset{i}{\sum }{\bar{p}}_{i}L_{reg}\left(t_{i},{\bar{t}}_{i}\right)$$

where *p_i_* is the predicted probability of an anchor with index *i* being an object in mini-batch. The ground-truth label ${\bar{p}}_{i}$ is 1 if the anchor is positive and is 0 if the anchor is negative. Moreover, $t_{i}$ is a vector representing the four coordinates of the predicted bounding box, and ${\bar{t}}_{i}$ is that of the ground-truth box associated with a positive anchor. The classification loss $L_{cls}$ is log loss over two classes (object versus not object) $L_{cls}\left(p_{i},{\bar{p}}_{i}\right)={\bar{p}}_{i}\log p_{i}-\left(1-{\bar{p}}_{i}\right)\log\left(1-p_{i}\right)$, $L_{reg}\left(t_{i},{\bar{t}}_{i}\right)=L_{1}\left(t_{i}-{\bar{t}}_{i}\right)$ where λ is the balancing parameter, $L_{1}$ is robust loss function, and $N_{cls}$ is normalized by the mini-batch size ($N_{cls}=256$) and the $N_{reg}\;$ is normalized by the number of anchor locations ($N_{reg}∼2400).$ The following section illustrates the proposed CNN that reduces the required computation cost and improves detection accuracy.

### 3.2. Proposed Dilated CNN

The traditional object detection algorithms include CNN where the image is convolved and then pooled. The pooling is used to increase the receptive field *R_F_* and reduce the amount of calculation. The receptive field *R_F_* is the part of the image that is defined by the filter size of the layer in the CNN [71]. This filter is used to extract the required features. Equation (2) shows the definition of the receptive field *R_F_*, where *k* is the size of the kernel and *d* is the space between each pixel in the convolution filter and called the dilation rate.

*R_F_* = *d*(*k* − 1) + 1 (2)

**Figure 2:**
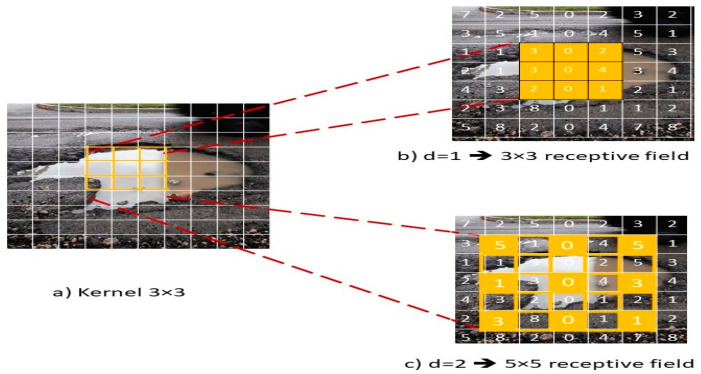
Dilated convolution: (**a**) original image with 3 × 3 kernel, (**b**) applying dilation rate of 1 with 3 × 3 kernel produces receptive field with size 3 × 3, and (**c**) applying dilation rate of 2 with 3 × 3 kernel produces receptive field with size 5 × 5, as a result the filter could grab more contextual information.

To increase feature resolution, improve the quality of the training results, and decrease the required computational costs, this paper expands the receptive field *R_F_* by adding dilation rate *d* larger than one to the conv2D kernel through dilated convolution [72]. For example, if we use dilation rate of 1 and 3 × 3 kernel, it produces receptive field with size 3 × 3 that is the same as the standard convolution as shown in Figure 2b. However, if we use dilation rate *d = N,* as a result each input skips *N* pixels. Figure 2c shows an example of using 3 × 3 kernel having dilation rate *d* = 2, which is equivalent to the same field of view as 5 × 5 kernel. This shows that increasing the receptive field *R_F_* enabled the filter to grab more contextual information. Equation (3) shows the size of the output that can be calculated.

(3) $$\sigma =\left|\frac{g+2p-R_{F}}{s}\right|+1$$

where *g* × *g* is the input with a dilation factor, padding, and stride of *d*, *p*, and *s*, respectively. Finally, using several receptive fields with different σ sizes enable us to grab valuable features in the scene area having different scales. In conclusion, dilated convolutions support exponentially enlarging the receptive fields without missing any coverage or resolution [72].

The VGG16 [65] network was designed for large scale image classification. VGG16 has 5 blocks of 13 convolutional layers and 3 fully connected layers and has a total of 138 million parameters. The convolution layers use 3 × 3 kernel size. Therefore, they have a very small receptive field *R_F_* = 3 × 3 to capture the smallest size notion of left/right, up/down, center. Spatial pooling is carried out by adding five max-pooling layers that follow some of the convolutional layers. Each maxpool layer has a 2 × 2 kernel size with a stride of two. The use of multiple pooling of high-level features lead to loss of some details and features. Therefore, this paper proposes a modified version of VGG16 (MVGG16) to generate high quality training results and reduce the required computation cost.

**Figure 3:**
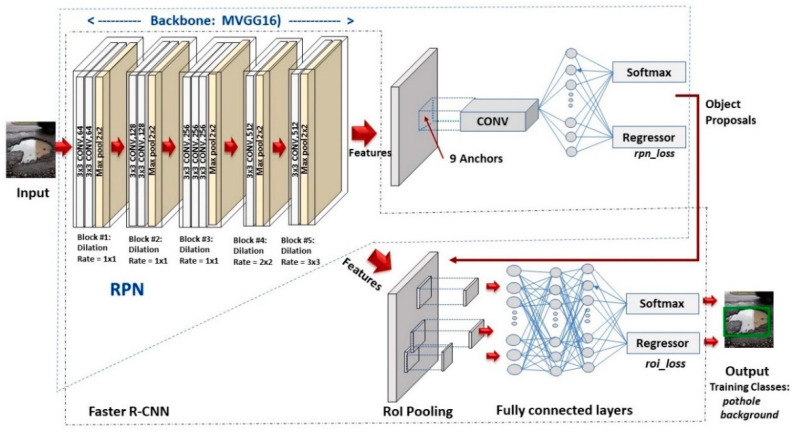
Faster R-CNN with modified VGG16 (MVGG16).

The proposed MVGG16 has five blocks including nine convolution layers and five maxpool layers as shown in Figure 3. It has total of 5.28 million parameters. The first seven convolution layers used 3 × 3 kernel size and dilation rate 1 × 1. The last two convolution layers use 3 × 3 kernel size and dilation rates 2 × 2 and 3 × 3, respectively, as shown in Figure 3. Similar to VGG16, in all convolution layers, we used rectified linear units (*ReLUs*) as activation functions. To generate region proposals, we slide a small network over the MVGG16 map output by the last shared convolutional layer. This small network takes as input a 3 × 3 spatial window of the input convolutional feature map. Each sliding window is mapped to a lower-dimensional feature (512-d for MVGG16, with *ReLU* following). This feature is fed into two sibling fully connected layers, a box-regression layer and a box-classification layer. In summary, the MVGG16 modifies VGG16 by removing f our convolution layers from the last two blocks and use different dilation rates, as shown in Figure 3.

### 3.3. YOLOV5

Redmon et al. [40] proposed an object detection algorithm “YOLO” that was claimed to be more usable in real time than the prevailing algorithms because of its speed in detecting objects. An input image is divided into *S × S* grid cells and some grid cells are responsible for detecting an object present in the image, i.e., only the ones where the center of the bounding box is in the cell. There were *β* bounding boxes, and confidence scores for those boxes were predicted for each grid cell. The bounding box prediction is composed of five components: (*x*, *y*, *w*, *h*, *c*), where (*x*, *y*) coordinates give the center of the box, (*w*, *h*) give the width and height of the box, and c gives the confidence score of the box. There are in total *S × S × β × 5* outputs for an image input. The presence or absence of a pothole can be ascertained from the confidence score. As in [40], we define confidence score as where Pr(Object) is the probability of pothole appearing in a grid cell and *IoU* is the intersection of union between the ground truth and the predicted boxes as shown in Equation (10). If no pothole exists in that box, the confidence score should be zero. The *GIoU* metric in Equation (4) is used to evaluate how close the prediction bounding box (*A*) is to the ground truth box (*B*), where $A,B\subseteq S\in {\mathbb{R}}^{𝓃}\;$ and object shape (*C*), $C\subseteq S\in {\mathbb{R}}^{𝓃}$ [73].

(4) $$GIoU=IoU-\frac{\left|C\backslash (A\cup B)\right|}{\left|C\right|}$$

The sum of the following Equations (5)–(8) is the loss function that penalizes bounding box, coordination error, and classification error [40].

(5) $$\lambda_{coord}\overset{S^{2}}{\underset{i=0}{\sum }}\overset{\beta }{\underset{j=0}{\sum }}{𝕝}_{ij}^{obj}{\left(x_{i}-{\hat{x}}_{i}\right)}^{2}+{\left(y_{i}-{\hat{y}}_{i}\right)}^{2}$$

Equation (5) computes the loss related to the predicted bounding box position (*x*, *y*) and the actual position $\left(\hat{x},\;\hat{y}\right)$ from the training data. This function computes a sum over each bounding box predictor (*j* = 0 … *β*) of each grid cell (*i* = 0 ... *S^2^*) ${𝕝}_{i}^{obj}$, where ${𝕝}_{i}^{obj}$ implies that object appears in cell *i* and ${𝕝}_{ij}^{obj}$ indicates that *j*th bounding box predictor in cell *i* is responsible for that prediction. Equation (6) computes loss related to the coordination error of the predicted box width/height. It is like Equation (5), but the square root is used to reflect that the small deviations in large boxes matter less than those in small boxes. Thus, we can predict the square root of the bounding box width and height instead of the width and height directly.

(6) $$\lambda_{coord}\overset{S^{2}}{\underset{i=0}{\sum }}\overset{\beta }{\underset{j=0}{\sum }}{𝕝}_{ij}^{obj}\left[{\left(\sqrt{w_{i}}-\sqrt{{\hat{W}}_{i}}\right)}^{2}+{\left(\sqrt{h_{i}}-\sqrt{{\hat{h}}_{i}}\right)}^{2}\right]$$

Equation (7) computes the loss related to the classification error based on the confidence score for each bounding box predictor. Here, *C* is the confidence score and *Ĉ* is the intersection over union of the predicted bounding box with the ground truth; ${𝕝}_{j}^{obj}$ is equal to one when there is an object in the cell, and 0 otherwise, and ${𝕝}_{i}^{noobj}$ is the opposite.

(7) $$\overset{S^{2}}{\underset{i=0}{\sum }}\overset{\beta }{\underset{j=0}{\sum }}{𝕝}_{ij}^{obj}{\left(C_{i}-{\hat{C}}_{i}\right)}^{2}+\lambda_{noobj}\overset{S^{2}}{\underset{i=0}{\sum }}\overset{\beta }{\underset{j=0}{\sum }}{𝕝}_{ij}^{noobj}{\left(C_{i}-{\hat{C}}_{i}\right)}^{2}$$

The *λ* parameters seen in Equations (5)–(7) are used to differently weight the loss functions to improve the model stability ($\lambda_{coord}=5,\;\lambda_{noobj}=0.5)$. Equation (7) computes the classification loss as normal sum-squared error for classification, except for the ${𝕝}_{}^{obj}$ term.

**Figure 4:**
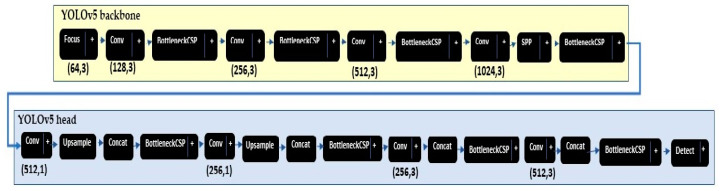
YOLOv5 small model as displayed by Netron app [77].

After the official release of YOLO in 2016, there have been four more revisions: YOLOv2 (darknet-19 backbone) [66], YOLOv3 (darknet-53 backbone) [67], YOLOv4 (e.g., CSP Darknet53 backbone) [74], YOLOv5 [68], and You Only Learn One Representation (YOLOR) [75]. The YOLOv5 was developed and published by Glenn Jocher, Ultralytics LLC in 2020 as a GitHub repository [68]. There are four major models of YOLOv5 based on the complexity of architecture, i.e., XS, S, M, and L. This paper provides a performance analysis of YOLOv5 Large (Y_l_), Medium (Y_m_), and Small (Y_s_) models. The YOLOv5 models [68] includes two main parts: the model backbone and the model head, as shown in Figure 4. First, to extract important features from the given input image the model backbone is used. YOLOv5 used ResNet101 to develop the cross-stage partial (CSP) bottleneck that reduces the network parameters, extracts the informative features from an input image [76], and reuses the captured features. Second, YOLOv5 developed the final detection part (model head) for feature aggregation. It is responsible to generate the final output vectors including bounding boxes, confidence scores, and class probabilities. In YOLOv5, the final detection layers used the *Sigmod* activation function; however, the middle or hidden layers used *Leaky ReLU* activation functions. Finally, to filter the false predictions, in this paper we ignore any prediction that has a confidence score lower than 0.5. The YOLOv5 used the k-mean clustering algorithm with different *k* values to automatically determine the best anchor boxes for that dataset and use them during training. The YOLOv5 calculates a total loss function from regression loss *box_loss* (based on *GIoU*; Equations (4)–(6)), *obj_loss* (based on *IoU*; Equations (7) and (10)) and classification loss cls_loss. In this paper, the *cls_loss* equals zero because our problem is to only to detect objects.

## 4. Results

This section comprises the description of the experimental environment and visualization of performance metrics performed by trained models on the pothole dataset.

### 4.1. Setup

The machine used for training was running Windows 10 and was embedded with Intel Core i5 CPU, GPU of NVIDIA RTX 2080(8GB), and 16GB memory. Different packages of Python3 such as OpenCV [78], PyTorch [79], Cudatoolkit [80], NumPy [81], and Tensorboard [82] were installed. We used momentum of value 0.843 and weight decay of 0.00036 for YOLOv5, whereas none were used for Faster R-CNN. YOLOv5 dynamically calculates the anchor size and aspect ratios; however, in Faster R-CNN we set anchor sizes and aspect ratios as shown in Table 2. Furthermore, the parameters used for training are listed in Table 2. It is noted that Faster R-CNN was able to converge in a smaller number of epochs (100 epochs) than YOLOv5 (1200 epochs) to generalize the model. In addition, we used mini batch size to increase the Faster R-CNN and YOLOv5 model’s accuracy and to efficiently utilize the memory of the GPU.

#### 4.1.1. Dataset Preparation

**Figure 5:**
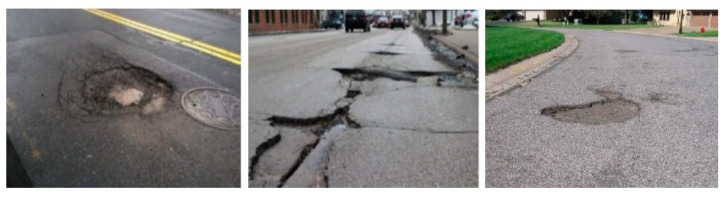
Sample images from potholes dataset.

There is no online benchmark potholes dataset available and a few publicly available official datasets for pothole detection. Therefore, in this work we accumulated pothole images from multiple sources (MakeML [83] and Roboflow [84]), and we used smartphone video cameras attached to vehicle windshields to collect other images from roads in Carbondale, IL. Out of the total images, 665 images with 2139 potholes were used for training, 183 images with 327 potholes for validating the model, and 92 images for testing the generated model. The images in the dataset include several potholes with different shape, area, and depth, as shown in Figure 5. We used *LabelImg* [85], an open-source graphical annotation tool, to label our images in PASCAL/VOC format and later converted them into .txt format for YOLOv5 and .csv format for Faster R-CNN. The size of images used ranged from 14KB to 960KB and the shape of images ranged from 270 × 150 to 3264 × 1836.

#### 4.1.2. Dataset Augmentation

**Figure 6:**
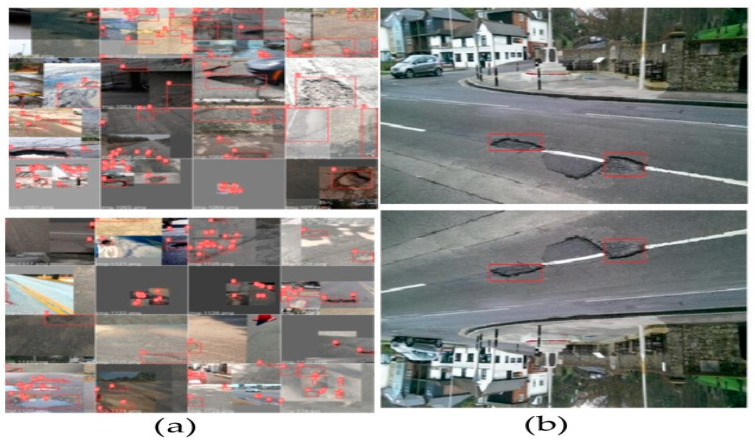
Data augmentations: (**a**) Mosaic YOLOv5, (**b**) Faster R-CNN.

The limited size of the dataset can lead to over-fitting. However, deep learning models demand a satisfactory amount of data to generate accurate results [86]. Therefore, we have applied augmentation techniques to avoid over-fitting as well as to gain advantage of regularization. Various parameters such as scaling, color adjustments, rotation, and mosaic augmentation, etc., were used for augmentation. Mosaic augmentation in YOLOv5 is one of the peculiar types of augmentation ever used before. It combines multiple images cropped randomly to form a grid as shown in Figure 6a. YOLOv5 authors have maintained their own code for augmentation, whereas Albumentations [87] library was used for augmentation in Faster R-CNN. We have used a scale factor of 0.5, shear of 0.5, flip up-down of 0.2 and flip left-right of 0.5, mosaic of 1, and translation of 0.1 for augmenting in training YOLOv5. Similarly, with Faster R-CNN, flip of 0.5, vertical flip of 0.00856, and horizontal flip of 0.5 were used. Examples of images augmented and used in training model generated by Faster R-CNN are shown in Figure 6b. Images in YOLOv5 were resized by scaling one of the larger image’s dimensions to 640 and another dimension was rescaled maintaining the aspect ratio. However, no image resizing was performed for Faster R-CNN.

### 4.2. Performance Evaluation Mertics

In object detection, metrics such as precision, recall, accuracy, and mean average precision (*mAP*) are used to evaluate the performance of the prediction model. These metrics can be used to compare the performance of different object detection algorithms on the same dataset. Precision measures the accuracy of the model in predicting potholes, whereas the accuracy is the ratio of correct detection to the total images used for testing. Recall measures the performance of the model in finding all potholes in the images. All these measures are directly affected by the *IoU*. The confidence threshold ω  is defined as the ratio of intersection of ground truth and prediction area to the union of ground truth and prediction area. The ω is used to distinguish whether detection is valid or invalid (commonly used ω=0.5). The metrics discussed are formulated by Equations (8)–(13) below:

(8) $$Precision=\frac{T_{P}}{All\;detections}=\frac{T_{P}}{T_{P}+F_{P}}$$

(9) $$Recall=\frac{T_{P}}{All\;ground\;truths}=\frac{T_{P}}{T_{P}+F_{N}}\;$$

(10) $$IoU=\frac{A\cup B}{A\cap B}\;$$

(11) $$Accuracy=\frac{T_{P}+T_{N}}{T_{P}+T_{N}+F_{P}+F_{N}}\;$$

(12) $$AP@\omega =\int_{0}^{1}p(r)dr$$

(13) $$mAP@\omega =\frac{1}{N}\sum_{i=1}^{N}AP_{i}$$

where *A* is the prediction bounding box and *B* is the ground truth box, respectively, $A,B\subseteq S\in {\mathbb{R}}^{𝓃}$ [73] and *T_P_* is true positives, *T_N_* is true negatives, *F_N_* is false negatives, and *F_P_* stands for false positives. Because there are a large number of instances that should not be detected as objects, the *T_N_* metric does not apply in object detection. Using frame-based constraints, if the bounding box region contains the foreground object (pothole), then the frame demonstrates true positive when IoU≥ω. If the object is not present inside the bounding box, then frame is considered as false positive where IoU<ω. The frame shows false negative if target object missed by the bounding box (i.e., ground-truth missed by the model). The precision-recall (*PR*) curve plots precision as a function of recall. It depicts the trade-off between the precision and recall for varying confidence values for the model detections. The average precision (*AP*@ω) is the area under the *PR* curve as shown in Equation (12), where precision and recalls are always between 0 and 1. The mean average precision (*mAP@*ω) is the average of the *AP@*ω calculated for all the classes as shown in Equation (13). It is used to determine the accuracy of a set of object detections from a model when compared to ground-truth object of the dataset.

## 5. Object Detection Results and Discussion

**Figure 7:**
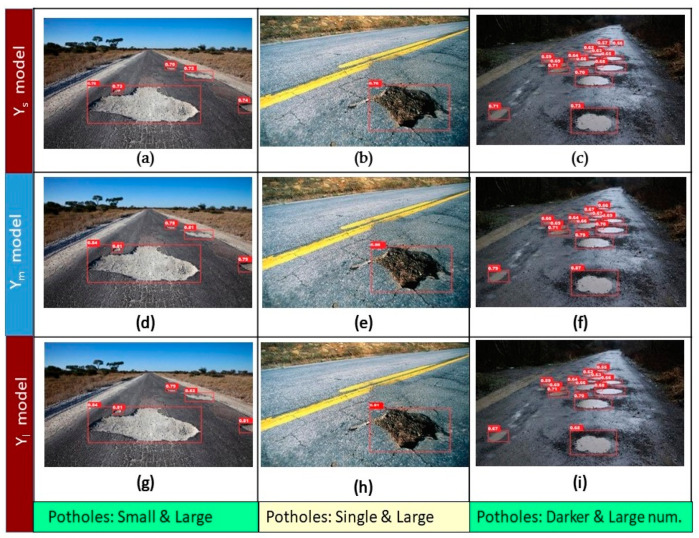
Detection results using YOLOv5: (**a**–**c**) small; (**d**–**f**) medium; (**g**–**i**) large models.

**Figure 8:**
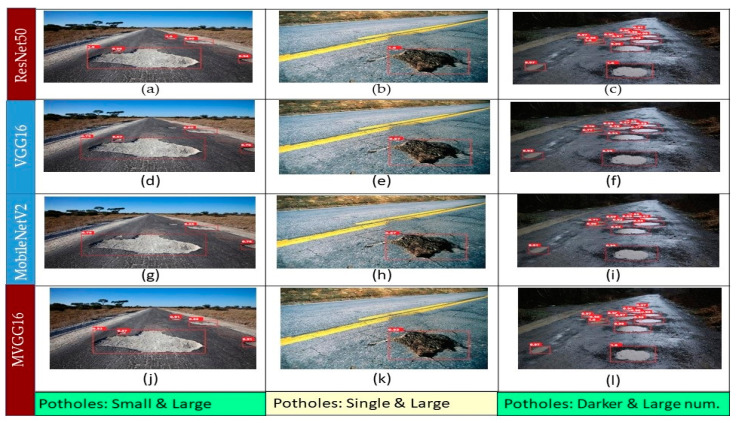
Detection results using Faster R-CNN with different backbones: (**a**–**c**) ResNet50; (**d**–**f**) VGG16; (**g**–**i**) MobileNetV2; (**j**–**l**) MVGG16.

The comparison of results achieved by the YOLOv5 models and Faster R-CNN models in sample images of different daylight conditions and with different numbers of potholes are shown in Figure 7 and Figure 8, respectively. Figure 7 shows the prediction results of YOLOv5 Y_s_ (a–c), Y_m_ (d–f), and Y_l_ (g–i) models. The images in the right column are comparatively darker than the other images, we can see that all the models are able to detect the potholes correctly, but the Y_l_ model shows better confidence scores on average than the other two models. The images in the middle column include a single and bigger pothole; all the models were able to detect it well, but the confidence score Y_m_ shows the highest score (0.88), followed by the Y_l_ model. Each model has different detection results for images in the rightmost column, which contains a relatively higher number of potholes. The Y_s_ model was able to detect a higher number of potholes than the other models. The Y_m_ and Y_l_ models detected same number of potholes. In summary, as shown in Figure 7, the Y_l_ model can easily detect visible and bigger potholes with satisfactory confidence score, the Y_m_ model is able to detect potholes with higher a confidence score, and the Y_s_ model is able to detect the largest number of potholes.

**Figure 9:**
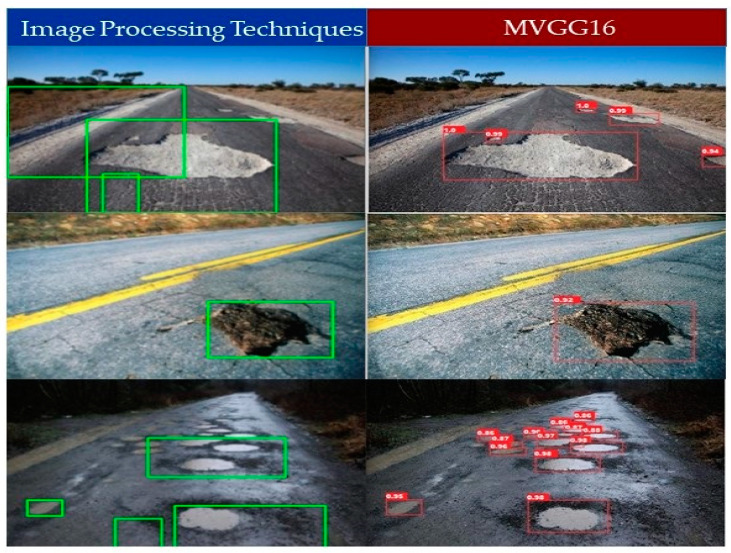
Pothole detection results: left column pothole detection using image processing techniques [24] and right column pothole detection using Faster R-CNN with backbone MVGG16.

Figure 8 shows the prediction results of Faster R-CNN with different backbones for the same images used for the YOLOv5 model comparison. Figure 8a–l shows the results of Faster R-CNN with ResNet50(FPN), VGG16, MobileNetV2, and MVGG16 backbones, respectively. The left column in Figure 8 shows that ResNet50 successfully detect all the potholes with the highest confidence score (99%), whereas VGG16 and MobileNetV2 successfully detect only four and three potholes, respectively. Figure 8g shows that MobileNetV2 was able to detect bigger potholes only and fails to detect small/far-away ones, as shown in Figure 8h,i. MVGG16 was able to detect potholes with a higher confidence score than VGG16 and MobileNetV2. The middle column in Figure 8 depicts that ResNet50 makes a perfect prediction with a perfect bounding box and confidence score. However, VGG16 and MobileNetV2 fail to detect the bounding box accurately, although the confidence score is above 90% for both. The proposed MVGG16 successfully detected the perfect bounding box with a high confidence score. Each of the models have different detection results in images in the right column in Figure 8. ResNet50 detects bigger and visible potholes with a relatively better confidence score than the rest of the models. We can see that VGG16 has the worst confidence score in this image and MobileNetV2 is unable to detect one of the potholes. However, MVGG16 is able to detect the potholes with a better confidence score than VGG16. Overall, it is obvious that ResNet50 is the best among the four models in accuracy. However, MVGG16 outperformed all models in terms of inference speed. In addition, we developed the image processing techniques in [24] to detect potholes and compared the results with the Faster R-CNN with MVGG16 as shown in Figure 9. It shows that the image processing techniques failed to detect potholes due to the variation of the pothole size.

### Comparison of YOLOv5 and Faster R-CNN (MVGG16)

**Figure 10:**
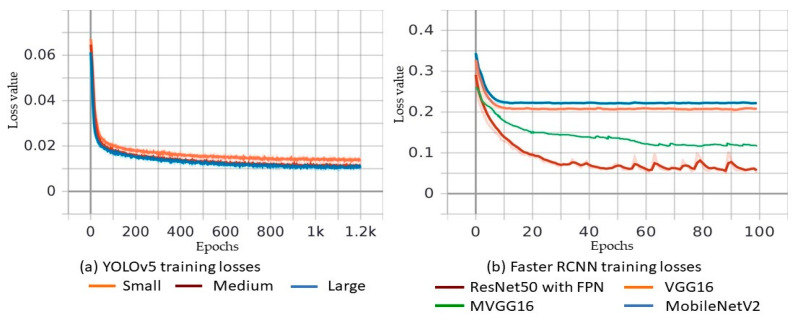
(**a**) Training loss values for YOLOv5 and (**b**) training loss values for Faster R-CNN with four backbones: ResNet50 with FPN, VGG16, MVGG16, and MobileNetV2.

**Figure 11:**
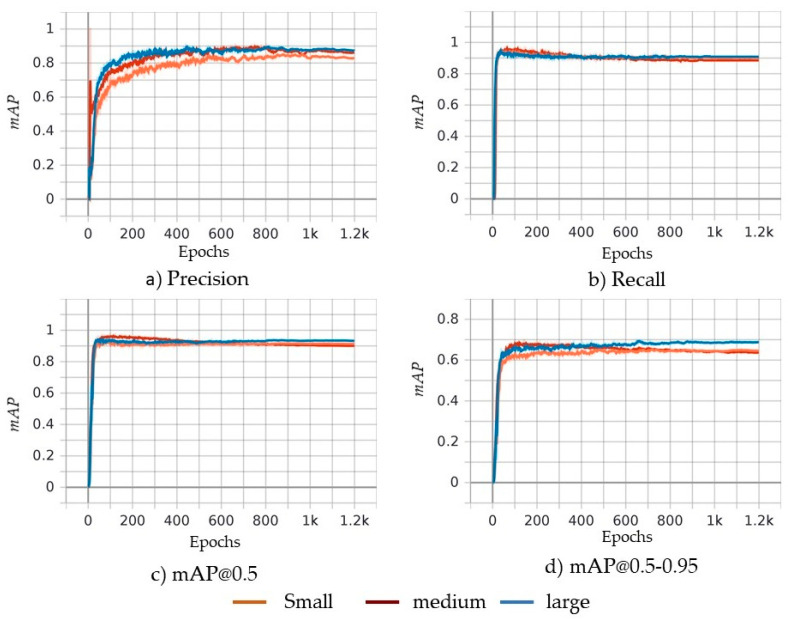
Training comparison of YOLOv5 Y_s_, Y_m_, and Y_l_ models using different metrics: (**a**) precision value of Y_s_, Y_m_, and Y_l_ in different epochs of training, (**b**) recall value of Y_s_, Y_m_, and Y_l_ in different epochs of training, (**c**) mAP@0.5 of Y_s_, Y_m_, and Y_l_ in different epochs of training, and (**d**) mAP@0.5–0.95 of Y_s_, Y_m_, and Y_l_ in different epochs of training.

In the previous section, we compared the inference of models in three sample images. This section presents the comparison of the training metrics such as precision, recall, mAP, and loss values of the algorithms along with the other comparison criteria that directly affect the usability of models in real-time situations. The training loss values for different models of YOLOv5 and Faster R-CNN are shown in Figure 10a and Figure 10b, respectively. The training loss value of YOLOv5 [40] is obtained using Equations (5)–(8). Loss values graph for the Y_s_, Y_m_, and Y_l_ models show that the nature of curve is same, but model Y_l_ has a relatively lower value of loss, followed by Y_m_ and Y_s_ models, as shown in Figure 10a. Faster R-CNN [43] uses multi-task loss of the joint training for both classification and bounding-box regression values as shown in Equation (1), where classification loss represents category loss, and regression loss represents bounding box location loss. Figure 10b depicts the loss values of Faster R-CNN with ResNet50, MVGG16, VGG16, and MobileNetV2 backbones. ResNet50 outperforms the rest of the backbones because it has half the loss values that the other models have. ResNet50 is followed by MVGG16, VGG16, and MobileNetV2 for lower training loss values. Figure 11 contains a comparison of YOLOv5 models based on different metrics discussed in previous section. The nature of the curve of all models is almost similar for all accuracy metrics except for some differences in values. Figure 11a shows the precision value of different models in different epochs of training. The precision value of large model Y_l_ is greater, followed by the Y_m_ and Y_s_ model. It is similar for recall and mAP@0.5–0.95 as shown in Figure 11b and Figure 11d, respectively. However, the Y_s_ model surpassed the Y_m_ model at the end of training in the value of mAP@0.5 as shown in Figure 11c. In conclusion, the YOLOv5 Y_l_ model stays on top in accuracy values followed by Y_m_ and Y_s_ models, and it achieved high precision 86.4% and YOLOv4 achieved 85% [37]. The summary of comparison of various models discussed in this research is tabulated in Table 3. We can see that ResNet50 has the highest *precision* value followed by Y_m_, Y_l_ and the proposed MVGG16, whereas MobileNetV2 is last. Similarly, training loss value for Y_s_ model is smaller compared to all models of YOLOv5 and Faster R-CNN. The proposed MVGG16 reduced the VGG16 training loss by approximately 40%. The ResNet50 has the largest value of mAP@0.5–0.95 at 64.12%, whereas MobileNetV2 has the worst mAP@0.5–0.95 value. It is noticed that MVGG16 improved the VGG16′s mAP@0.5–0.95 value by 10%. For two image resolutions, the Y_s_ model has the best value for inference speed as expected and VGG16 has the worst value. The proposed MVGG16 was able to improve the VGG16 inference speed by 58.7%. The Y_s_ model showed the lowest training time per epoch, but it required more epochs to converge. However, MobileNetV2 converged in just 100 epochs with 8000 s for total training time, which was the fastest. When it comes to final model size, all the Faster R-CNN models have bigger sizes than th eYOLOv5 models. Table 3 shows that the proposed MVGG16 produces the smallest model in size for all Faster R-CNN. The Y_s_ model has the smallest size which of only14.8MB; however, MVGG16 generates the smallest Faster R-CNN model. The MVGG16 reduces the VGG16 models’ size by approximately 18.8%, as shown in Table 3. The models listed in Table 3 achieved superior results compared with the image processing techniques that achieved precision of 81.8 in [22] and 88.0 in [58] with detection speed ≈ 0.2 s, which is not suitable for real-time potholed detection. In addition, we tested You Only Learn One Representation (YOLOR) [75], which integrates implicit knowledge that is obtained from shallow layers and explicit knowledge that is obtained from deep layers. YOLOR generates a model that could contain a general representation to enable sub-representations appropriate for various tasks.

Table 4 lists the comparison of YOLOv5 small, Faster R-CNN with MVGG16 backbone, YOLOR-P6, and YOLOR-W6. The training of the YOLOR-W6 and YOLOR-P6 require large GPU memory, approximately 6.79GB and 11.3GB per epoch, respectively. The mAP@0.5–0.95 of the YOLOv5 (Y_s_) is the largest value, 58.9%, followed by Faster R-CNN with MVGG16 backbone and YOLOR-W6, respectively, whereas YOLOR-P6 stays last as shown in Table 4. Moreover, the YOLOv5 (Y_s_) has the smallest model size, 14.8MB, followed by Faster R-CNN with MVGG16 backbone and YOLOR-P6, respectively, whereas YOLOR-W6 generated the largest model size. In conclusion, the proposed Faster R-CNN MVGG16 backbone developed a deep CNN having different dilated layers to increase receptive fields. Thus, the generated model succeeds to balance its accuracy and inference speed because it: (1) reduces the required computations in CNN backbone (e.g., Resnet50) and (2) improves the model accuracy compared with other backbones such as VGG16 and MobileNet.

## 6. Conclusions and Future Work

Considering the needs to detect potholes in roads accurately and in real-time, this paper developed efficient CNN models. The conducted experiments in this paper used a dataset that includes pothole images that were collected in different daylight conditions, different road conditions, and with different shapes and sizes. The pothole dataset was trained with ten different CNNs: three variations of YOLOv5 (Y_l_, Y_m_, and Y_s_), two variations of YOLOR, and Faster R-CNN with five different backbones (ResNet50, VGG16, MobileNetV2, InceptionV3 and the proposed CNN called MVGG16). Experiments show that Faster R-CNN ResNet50 has the highest precision of 91.9% followed by Y_m_, Y_l_, and the proposed MVGG16 whereas MobileNetV2 was last. The Y_s_ model is the fastest model to predict potholes followed by Y_l_, Y_m_, MobileNetV2, MVGG16, InceptionV3, and ResNet50, and VGG16 was slowest. In addition, the results show that MVGG16 improves the precision and shortens the inference speed compared with VGG16. However, the Y_s_ model is the fastest one in detecting all the potholes in high resolution and low-resolution images in 0.009 s, but the MVGG16 model outperforms the precision of Y_s_ by 4.67%. In addition, the experiments show that largest generated model in size is MobileNetV2, and it achieves the lowest precision value with 63.1% for pothole detection. The VGG16 produces the slowest model with a detection speed of 0.11s per image. The proposed MVGG16 is able to outperform the detection time of the VGG16 by 58.7%. Analyzing the inference results and accuracy metrics, we suggest using the Y_s_ model in real-time scenarios like embedding them in vehicles, because it has a satisfactory detection speed. Similarly, Faster R-CNN with ResNet50 can be used with a more sophisticated hardware setup in scenarios where accuracy is the major concern. In addition, MVGG16 generates a model with smaller size than Faster R-CNN with ResNet50. Therefore, we suggest using the MVGG16 if model size is a concern when using Faster R-CNN. There were some pothole images that were taken in extreme weather conditions like snow, some images were in very bad road conditions, and some images were taken with unusual camera angles, which directly affected the performance of models in validation sets. In our future research, we will develop a sustainable model to address these extreme conditions. The accuracy can further be increased by using training images that are taken from vehicle cameras in an angle that the model will use later to predict and by adding more variation to the training images. Furthermore, as an extension of this research, the depth of potholes and the distance (in meters) may also be estimated using calibrated stereo cameras.

## Figures and Tables

**Table 1:** Pothole detection approaches.

Approaches	Limitations
**Sensor-based** [8,9,10,11,12]	- Specific devices are needed - Sensors may get damaged due to road conditions - False negative potholes in the center of a lane: - False positive: road-joints detected as potholes - Lacks information: Area and shape of potholes
**3D reconstruction** Laser [13,14,15], Stereo vision [16,17,18,19]	- Expensive 3D laser scanner - High computational efforts to reconstruct surface - Repetitive camera alignments are needed
**Image processing** Images [21,22,23,24] Videos [21,25,26,27,28]	- Require adjusting several parameters and steps for different road conditions - High computational complexity - Not suitable for real-time
**Model-based** [29,30,31,32,33,34,35,36,37] Machine learning	- Need large training data - Manual feature extraction - Shallow models
Deep learning	- Require balancing the model’s accuracy and detection time

**Table 2:** Training parameters for YOLOv5 and Faster R-CNN.

Parameters	YOLOv5	Faster R-CNN		
		ResNet50 (FPN)	VGG16, MVGG16	MobileNetv2, InceptionV3
Batch Size	Y_L_ = 8, Y_m_, Y_s_ = 16	2	2	2
Epochs	1200	100	100	100
Learning Rate	0.0032	0.005	0.0001	0.0001
Optimizer	SGD	SGD	Adam	Adam
Anchor Sizes	Dynamic	32, 64, 128, 256, 512	8, 16, 32, 64, 128, 256, 512	8, 16, 32, 64, 128, 256, 512

**Table 3:** Comparison of YOLOv5 and Faster R-CNN performance.

Metrics	YOLOv5			Faster R-CNN [43]				
	Y_l_	Y_m_	Y_S_	ResNet50 (FPN)	VGG16	MVGG16	Mobile-Net V2	Inception V3
Precision (P)	86.43%	86.96%	76.73%	91.9%	69.8%	81.4%	63.1%	72.3%
Training Loss	0.015	0.017	0.020	0.065	0.226	0.136	0.209	0.194
Mean Average Precision (mAP@0.5–0.95)	63.43%	61.54%	58.9%	64.12%	35.3%	45.4%	30.5%	32.3%
Inference speed: Image resolution (1774 × 2365)	0.014 s	0.012 s	0.009 s	0.098 s	0.114 s	0.047 s	0.036 s	0.052 s
Inference speed: Image resolution (204 × 170)	0.018 s	0.013 s	0.009 s	0.065 s	0.119 s	0.052 s	0.032 s	0.056 s
Training time/epoch	26 s	16 s	12 s	124 s	173 s	105 s	80 s	95 s
Total training time	31,200 s	19,200 s	14,400 s	12,400 s	17,300 s	10,500 s	8000 s	9500 s
Model Size (MB)	95.3	43.3	14.8	165.7	175.5	134.5	329.8	417.2

**Table 4:** Comparison of YOLOv5 small, Faster R-CNN with MVGG16, and YOLOR models.

	YOLOv5 (Y_s_)	Faster R-CNN with MVGG16	YOLOR- P6	YOLOR- W6
Training (batch size, epochs, learning rate)	(16, 1200, 0.0032)	(2, 100, 0.0001)	(8, 1200, 0.01)	(8, 1200, 0.01)
Training Loss	0.020	0.136	0.0170	0.015
*mAP*@0.5-0.95	58.9%	45.4%	43.2%	44.6%
Inference speed: Image resolution (1774 × 2365)	0.009 s	0.047 s	0.03 s	0.032 s
Inference speed: Image resolution (204 × 170)	0.009 s	0.052 s	0.03 s	0.032 s
Model Size (MB)	14.8	134.5	291.8	624.84

## Data Availability

Publicly available datasets were used in this study.

## References

[1] Solanke V.L., Patil D.D., Patkar A.S., Tamrale G.S., Kale P.A.G. (2019). Analysis of existing road surface on the basis of pothole characteristics. Glob. J. Res. Eng..

[2] T.A.A. Association Pothole Damage Costs Drivers $3 Billion Annually Nationwide. http://news.aaa-calif.com/news/pothole-damage-costs-drivers-3-billion-annually-nationwide.

[3] The Economic Times Supreme Court Takes Note of 3597 Deaths Due to Pothole-Related Accidents in 2017. https://economictimes.indiatimes.com/news/politics-and-nation/supreme-court-takes-note-of-3597-deaths-due-to-pothole-related-accidents-in-2017/articleshow/65858401.cms.

[4] (2018). S. Portal. City of San Antonio: Potholes. https://www.sanantonio.gov/PublicWorks/FAQs/Streets.

[5] San Antonio (2019). “San Antonio Fiscal Year 2019 2nd Quarter Report: Providing Services/Measuring Results” San Antonio. https://www.sanantonio.gov/Portals/0/Files/budget/FY2019/FY2019-six-plus-six-Performance-Measures.pdf.

[6] Portal C. (2021). City of Chicago Data Portal. https://data.cityofchicago.org/.

[7] Bureau of Transportation Statistics (2018). Road Condition. https://www.bts.gov/road-condition.

[8] Yu B.X., Yu X. Vibration-based system for pavement condition evaluation. Proceedings of the 9th International Conference on Applications of Advanced Technology in Transportation (AATT).

[9] Zoysa K.D., Keppitiyagama C., Seneviratne G.P., Shihan W. A public transport system based sensor network for road surface condition monitoring. Proceedings of the Workshop on Networked Systems for Developing Regions.

[10] Eriksson G. The pothole patrol: Using a mobile sensor network for road surface monitoring. Proceedings of the Sixth Annual International conference on Mobile Systems, Applications and Service.

[11] Mednis A., Strazdins G., Zviedris R., Kanonirs G., Selavo L. Real time pothole detection using android smartphones with accelerometers. Proceedings of the 2011 International Conference on Distributed Computing in Sensor Systems and Workshops (DCOSS).

[12] Tai Y.-C., Chan C.-W., Hsu J.Y.-J. Automatic road anomaly detection using smart mobile device. Proceedings of the Conference on Technologies and Applications of Artificial Intelligence (TAAI2010).

[13] Chang K., Chang J.R., Liu J.K. Detetction of pavement distress using 3D laser scanning. Proceedings of the ASCE International Conference on Computing in Civil Engineering.

[14] Yu X., Salari E. Pavement pothole detection and severity measurement using laser imaging. Proceedings of the International Conference on Electro/Information Technology.

[15] Moazzam I., Kamal K., Mathavan S., Usman S., Rahman M. Metrology and visualization of potholes using the Microsoft Kinect sensor. Proceedings of the 16th International IEEE Annual Conference on Intelligent Transportation Systems.

[16] Staniek M. (2017). Stereo vision techniques in the road pavement evaluation. Balt. J. Road Bridge Eng..

[17] He K., Zhang X., Ren S., Sun J. Deep residual learning for image recognition. Proceedings of the 2016 IEEE Conference on Computer Vision and Pattern Recognition (CVPR).

[18] Wang K.C.P. Challenges and feasibility for comprehensive automated survey of pavement conditions. Proceedings of the 8th International Conference on Applications of Advanced Technologies in Transportation Engineering.

[19] Hou Z., Wang K.C.P., Gong W. Experimentation of 3D pavement imaging through stereovision. Proceedings of the International Conference on Transportation Engineering.

[20] Zhang Z., Ai X., Chan C.K., Dahnoun N. An efficient algorithm for pothole detection using stereo vision. Proceedings of the IEEE International Conference on Acoustics, Speech and Signal Processing (ICASSP).

[21] Koch C., Brilakis I. (2011). Pothole detection in asphalt pavement images. Adv. Eng. Inform..

[22] Nienaber S., Booysen M.T., Kroon R. Detecting potholes using simple image processing techniques and real-world footage. Proceedings of the 34th Southern African Transport Conference (SATC 2015).

[23] Koch C., Jog G.M., Brilakis I. (2013). Ptholes detection with image processing and spectral clustering. J. Comput. Civ. Eng..

[24] Bhat A., Narkar P., Shetty D., Vyas D. (2018). Detection of Potholes using Image Processing Techniques. IOSR J. Eng..

[25] Jog G.M., Koch C., Golparvar-Fard M., Brilakis I. Pothole properties measurement through visual 2D recognition and 3D reconstruction. Proceedings of the ASCE Interntional Conference on Computing in Civil Engineering.

[26] Lokeshwor H., Das L.K., Sud S.K. (2013). Method for automated assessment of potholes, cracks and patches from road surface video clips. Procedia Soc. Behav. Sci..

[27] Kim T., Ryu S.-K. (2014). System and Method for Detecting Potholes based on Video Data. J. Emerg. Trends Comput. Inf. Sci..

[28] Muslim M., Sulistyaningrum D., Setiyono B. (2020). Detection andcounting potholes using morphological method from road video. AIP Conf. Proc..

[29] Lin J., Liu Y. Potholes detection based on svm in the pavement distress image. Proceedings of the 2010 Ninth International Symposium on Distributed Computing and Applications to Business, Engineering and Science.

[30] Yousaf M.H., Azhar K., Murtaza F., Hussain F. (2018). Visual analysis of asphalt pavement for detection and localization of potholes. Adv. Eng. Inform..

[31] Hoang N.-D. (2018). An artificial intelligence method for asphalt pavement pothole detection using least squares support vector machine and neural network with steerable filter-based feature exteraction. Adv. Civ. Eng..

[32] Hoang N.-D., Huynh T.-C., Tran V.-D. (2021). Computer Vision-Based Patched and Unpatched Pothole Classification Using Machine Learning Approach Optimized by Forensic-Based Investigation Metaheuristic. Complexity.

[33] Maeda H., Sekimoto Y., Seto T., Kashiyama T., Omata a.H. (2018). Road Damage Detection Using Deep Neural Networks with Images Captured through a Smartphone. Comput. Aided Civ. Infrastruct. Eng..

[34] Kotha M., Chadalavada M., Karuturi S.H., Venkataraman H. PotSense: Pothole Detection on Indian Roads using Smartphone Sensors. Proceedings of the 1st ACM Workshop on Autonomous and Intelligent Mobile Systems.

[35] Baek K., Byun Y., Song H. (2018). Pothole detection using machine learning. Adv. Sci Technol. Lett..

[36] Dharneeshkar J., Aniruthan S., Karthika R., Parameswaran L. Deep Learning based Detection of potholes in Indian roads using YOLO. Proceedings of the 2020 International Conference on Inventive Computation Technologies (ICICT).

[37] Shaghouri A.A., Alkhatib R., Berjaoui S. (2021). Real-Time Pothole Detection Using Deep Learning. arXiv.

[38] Silvister S., Komandur D., Kokate S., Khochare A., More U., Musale V., Joshi A. Deep learning approach to detect potholes in real-time using smartphone. Proceedings of the 2019 IEEE Pune Section International Conference (PuneCon).

[39] Szegedy C., Vanhoucke V., Joffe S., Shlens J., Wojan Z. Rethinking the inception architecture for computer vision. Proceedings of the 2016 IEEE Conference on Computer Vision and Pattern Recognition (CVPR).

[40] Redmon J., Divvala S., Girshick R., Farhadi A. You only look once: Unified, real-time object detection. Proceedings of the IEEE Conference on Computer Vision and Pattern Recognition (CVPR).

[41] Liu W., Anguelov D., Erhan D., Szegedy C., Reed S., Fu C.-Y., Berg A.C. SSD: Single shot multibox detector. Proceedings of the European Conference on Computer Vision.

[42] Ping P., Yang X., Gao Z. A Deep Learning Approach for Street Pothole Detection. Proceedings of the IEEE Sixth International Conference on Big Data Computing Service and Applications.

[43] Ren S., He K., Girshick R., Sun J. (2017). Faster r-cnn: Towards real-time object detection with region proposal networks. IEEE Trans. Pattern Anal. Mach. Intell..

[44] Siddique J.M., Ahmed K.R. (2021). Deep Learning Technologies to Mitigate Deer-Vehicle Collisions. Deep Learning and Big Data for Intelligent Transportation.

[45] Song H., Liang H., Li H., Dai Z., Yun X. (2019). Vision-based vehicle detection and counting system using deep learning in highway scenes. Eur. Transp. Res. Rev..

[46] Wu F., Duan J., Chen S., Ye Y., Ai P., Yang Z. (2021). Multi-Target Recognition of Bananas and Automatic Positioning for the Inflorescence Axis Cutting Point. Front. Plant Sci..

[47] Cao X., Yan H., Huang Z., Ai S., Xu Y., Fu R., Zou X. (2021). A Multi-Objective Particle Swarm Optimization for Trajectory Planning of Fruit Picking Manipulator. Agronomy.

[48] Ahmed K.R. (2021). Parallel Dilated CNN for Detecting and Classifying Defects in Surface Steel Strips in Real-Time. IntelliSys2021.

[49] Yang J., Li S., Wang Z., Dong H., Wang J., Tang S. (2020). Using Deep Learning to Detect Defects in Manufacturing: A Comprehensive Survey and Current Challenges. Materials.

[50] Chen M., Tang Y., Zou X., Huang K., Li L., He Y. (2019). High-accuracy multi-camera reconstruction enhanced by adaptive point cloud correction algorithm. Opt. Lasers Eng..

[51] Hautakangas H., Nieminen J. Data Mining for Pothole Detection Pro Gradu Seminar. https://slidetodoc.com/data-mining-for-pothole-detection-pro-gradu-seminar/.

[52] Liao P.S., Chen T.S., Chung P.C. (2001). A fast algorithm for multilevel thresholding. J. Inf. Sci. Eng..

[53] Matt G., Golub H., Von Matt U. (1991). Quadratically constrained least squares and quadratic problems. Numer. Math..

[54] Ai X., Gao Y., Rarity J.G., Dahnoun N. Obstacle detection using u-disparity on quadratic road surfaces. Proceedings of the 16th International IEEE Conference on Intelligent Transportation Systems (ITSC 2013).

[55] Umbaugh S.E. (2010). Digital Image Processing and Analysis: Human and Computer Vision Applications with CVIP Tools.

[56] Ouma Y.O., Hahn M. (2017). Pothole detection on asphalt pavements from 2D-colour pothole images using fuzzy c-means clustering and morphological reconstruction. Autom. Constr..

[57] Canny J. (1986). A Computational Approach to Edge Detection. IEEE Trans. Pattern Anal. Mach. Intell..

[58] Kim T., Ryu S.-K. (2015). Intelligent compaction terminal system for asphalt pavement in Korea. J. Emerg. Trends Comput. Inform. Sci..

[59] Ryu S.-K., Kim T., Kim Y.-R. (2015). Image-based pothole detection system for its service and road management system. Math. Probl. Eng..

[60] Liu L., Ouyang W., Wang X., Fieguth P., Chen J., Liu X., Pietikäinen M. (2020). Deep learning for generic object detection: A survey. Int. J. Comput. Vis..

[61] Li S., Li Y., Li Y., Li M., Xu X. (2021). YOLO-FIRI: Improved YOLOv5 for Infrared Image Object Detection. IEEE Access.

[62] Srivastava S., Divekar A.V., Anilkumar C., Naik I., Kulkarni V., Pattabiraman V. (2021). Comparative analysis of deep learning image detection algorithms. J. Big Data.

[63] Nguyen N.-D., Do T., Ngo T.D., Le D.-D. (2020). An Evaluation of Deep Learning Methods for Small Object Detection. J. Electr. Comput. Eng..

[64] Jiao L., Zhang F., Liu F., Yang S., Li L., Feng Z., Qu R. (2019). A Survey of Deep Learning-Based Object Detection. IEEE Access.

[65] Deng S.L.W. Very deep convolutional neural network-based image classification using small training sample size. Proceedings of the 3rd IAPR Asian Conference on Pattern Recognition (ACPR).

[66] Redmon J., Farhadi A. Yolo9000: Better, faster, stronger. Proceedings of theComputer Vision and Pattern Recognition (CVPR).

[67] Redmon J., Farhadi A. (2018). YOLOv3: An Incremental Improvement. arXiv.

[68] Jocher G. (2020). “Yolov5” LIC, Ultralytics. https://github.com/ultralytics/yolov5.

[69] Howard A.G., Zhu M., Chen B., Kalenichenko D., Wang W., Weyand T., Andreetto M., Adam H. (2017). Mobilenets: Efficient convolutional neural networks for mobile vision applications. arXiv.

[70] Lin T., Dollár P., Girshic R., Hariharan K.H.B., Belongie S. Feature pyramid networks for object detection. Proceedings of the 2017 IEEE Conference on Computer Vision and Pattern Recognition (CVPR).

[71] Luo W., Li Y., Urtasun R., Zemel R. (2017). Understanding the effective receptive field in deep. arXiv.

[72] Yu F., Koltun V. Multi-scale context aggregation by dilated convolutions. Proceedings of the International Conference on learning Representations (ICLR).

[73] Rezatofighi H., Tsoi N., Gwak J., Sadeghian A., Reid I., Savarese S. Generalized intersection over union: A metric and a loss for bounding box regression. Proceedings of the IEEE Conference on Computer Vision and Pattern Recognition.

[74] Bochkovskiy A., Wang C.-Y., Liao H.-Y.M. (2020). YOLOv4: Optimal Speed and Accuracy of Object Detection. arXiv.

[75] Wang C.-Y., Yeh I.-H., Liao H.-Y.M. (2021). You Only Learn One Representation: Unified Network for Multiple Tasks. arXiv.

[76] Wang C.-Y., Liao H.-Y.M., Wu Y.-H., Chen P.-Y., Hsieh J.-W., Yeh I.-H. Cspnet: A new backbone that can enhance learning capability of cnn. Proceedings of the 2020 IEEE/CVF Conference on Computer Vision and Pattern Recognition Workshops (CVPRW).

[77] Roeder L. (2021). Netron App Github. https://github.com/lutzroeder/netron.

[78] Bradski G. (2000). The OpenCV Library. Dr. Dobbs J. Softw. Tools.

[79] Ketkar N. (2017). Introduction to PyTorch. Deep Learning with Python.

[80] Kirk D. Nvidia cuda software and gpu parallel computing architecture. Proceedings of the 6th International Symposium on Memory Management—ISMM ’07.

[81] Oliphant T.E. (2015). A Guide to NumPy.

[82] Abadi M., Agarwal A., Barham P., Brevdo E., Chen Z., Citro C., Corrado G.S., Davis A., Dean J., Devin M. (2016). Tensorflow: Large-scale machine learning on heterogeneous distributed systems. arXiv.

[83] MakeML MakeML: Potholes Dataset. https://makeml.app/datasets/potholes.

[84] Chitholian A.R. Roboflow: Potholes Dataset. https://public.roboflow.com/object-detection/pothole.

[85] Lin T. (2021). Labelimg. https://github.com/tzutalin/labelImg.

[86] Chen X.-W., Lin X. (2014). Big Data Deep Learning: Challenges and Perspectives. IEEE Access.

[87] Buslaev A., Iglovikov V.I., Khvedchenya E., Parinov A., Druzhinin M., Kalinin A.A. (2020). Albumentations: Fast and flexible image augmentations. Information.

